# Associations of observer’s gender, Body Mass Index and internalization of societal beauty ideals to visual body processing

**DOI:** 10.1007/s00426-020-01471-5

**Published:** 2021-01-12

**Authors:** Valentina Cazzato, Elizabeth R. Walters, Cosimo Urgesi

**Affiliations:** 1 School of Psychology, Faculty of Health, Liverpool John Moores University, Liverpool, UK; 2 Division of Psychology, University of Bradford, Bradford, UK; 3 Department of Languages and Literatures, Communication, Education and Society, University of Udine, Udine, Italy; 4 Scientific Institute, IRCCS E. Medea, Bosisio Parini, Lecco Italy

## Abstract

We examined whether visual processing mechanisms of the body of conspecifics are different in women and men and whether these rely on westernised socio-cultural ideals and body image concerns. Twenty-four women and 24 men performed a visual discrimination task of upright or inverted images of female or male bodies and faces (Experiment 1) and objects (Experiment 2). In Experiment 1, both groups of women and men showed comparable abilities in the discrimination of upright and inverted bodies and faces. However, the gender of the human stimuli yielded different effects on participants’ performance, so that female faces, and male bodies appeared to be processed less configurally than female bodies and male faces, respectively. Interestingly, the reduction of configural processing for male bodies was significantly predicted by participants’ Body Mass Index (BMI) and their level of internalization of muscularity. Our findings suggest that configural visual processing of bodies and faces in women and men may be linked to a selective attention to detail needed for discriminating salient physical (perhaps sexual) cues of conspecifics. Importantly, BMI and muscularity internalization of beauty ideals may also play a crucial role in this mechanism.

**Supplementary Information:**

The online version contains supplementary material available at 10.1007/s00426-020-01471-5.

## Introduction

Information conveyed by both faces and bodies is of crucial importance for many aspects of social cognition. By looking at the body and face of our conspecifics, we can understand who these people are, how they are feeling, and what their next action is going to be (Reed & McIntosh, 2008; Slaughter, Stone, & Reed, 2004). More importantly, faces and bodies may reveal information about a partner’s health and reproductive potential. With these regards, it is crucial that humans are able to detect and assess those physical cues that indicate that one conspecific is fitter and with a better reproductive potential than another and then use these cues to select the partner who is most likely to enhance chances of successful reproduction (Buss, 2003; Thornhill & Gangestad, 1999).

Visual processing of human bodies and faces is normally thought to occur via the use of two different visual strategies, one based on the representation of the spatial relation among body parts in the context of the whole-body space (configural, or global processing; Reed, Stone, Grubb, & McGoldrick, 2006; Maurer, Grand, & Mondloch, 2002), and the second based on the analysis of the details of single body parts (local or feature-based processing). Whilst the feature-based visual strategy may be used in the discrimination of all objects, configural processing seems to be employed more in the discrimination of bodies and faces compared with other objects (e.g., Reed, Stone, Bozova, & Tanaka, 2003; Yovel, Pelc, & Lubetzky, 2010).

Traditionally, local vs. global visual processing have been investigated thanks to the use of several paradigms like for example the Navon paradigm (Navon, 1977), where participants are presented with hierarchical letters or figures that hold information at both the local and the global levels or, more relevant to our study, the inversion paradigm, which provides a measure of configural processing (Reed et al., 2003, 2006; Maurer et al., 2002) by estimating a drop of performance following presentation of inverted vs. upright body and face stimuli. These body inversion (BIE) and face inversion (FIE) effects seem to be explained by the fact that inversion disrupts the processing of configural information, and configural information is usually more important for the processing of bodies (and faces) than for the processing of objects (Reed et al., 2003, 2006).

However, a series of studies suggest that in clinical populations affected by body image disturbances (e.g., eating disorders and body dysmorphic disorder) this might not always be the case. Abnormal configural processing of body and face stimuli have been reported in both eating (Urgesi et al., 2014) and body dysmorphic disorders (Feusner et al., 2010a), and have also been linked with body image concern (BIC) in non-clinical populations (Beilharz, Atkins, Duncum, & Mundy, 2016; Mundy & Sadusky, 2014; Duncum, Atkins, Beilharz, & Mundy, 2016). In particular, Groves, Kennett and Gillmeister (2019) assessed configural processing strategies for faces and bodies in female and male adolescents aged 16–23 years; they also tested the relationship between the use of these strategies with BIC and self-objectification (i.e., best defined as a measure of whether an individual think about their bodies in terms of observable appearance, instead of competence). Although evidence of configural processing of faces and bodies was found in both women and men, the configural processing of bodies was partially reduced in young female participants, in particular in those at high risk of developing an eating disorder, and it was absent in women recovering from an eating disorder.

Interestingly, a series of work of Bernard, Gervais, Allen, Campomizzi and Klein (2012), Bernard, Gervais, Allen and Klein (2013) and Bernard, Gervais, Allen, Delmee and Klein (2015) measured the amount of BIE to ascertain the processing style associated with the perception of sexualized female and male targets. The authors found that, independently from the gender of the observer, sexualized women, but not sexualized men, portrayed in swimsuits were discriminated comparably well when presented in upright and inverted orientations. Furthermore, inverted female bodies were recognized better than inverted male bodies, suggesting that typically objectified female bodies are fragmented and recognized as a recollection of body parts, a piecemeal process which is typically seen during object recognition. As suggested by their recent hypothesis ‘Sexualised-body inversion hypothesis’ (SBIH), they concluded that sexualized women are processed locally as objects are, since their processing fails to show evidence of inversion effect.

Different attentional focus and gaze patterns during the visual exploration of human bodies have also been well documented (Arizpe, McKean, Tsao, & Chan, 2017). For example, it has been proven that participants tend to fixate on the chest and pelvic region longer than on the face region when scanning pictures depicting nude compared to clothed people (Nummenmaa, Hietanen, Santtila, & Hyönä, 2012). Similarly, Gervais, Holland and Dodd (2013) reported that participants focused longer on model women’s chests and waists than on faces when asked to evaluate their attractiveness. Moreover, this effect was particularly noticeable for model women with a more pronounced breast and lower waist-to-hip ratio, suggesting a role of body shape ideals on the attentional focus for body stimuli. The pattern of body exploration, however, was also influenced by the observer’s gender. In particular, both women and men tended to spend more time looking at the body of opposite than same-gender models; however, women tended to concentrate fixations onto the head area of male models, while men tended to fixate onto the bust and buttocks areas of female bodies. This is in keeping with the idea that the bust–waist ratio may provide a useful cue to the reproductive potential of a woman (Gervais, Vescio, Förster, Maass, & Suitner, 2012).

These works suggest that additional socio-cognitive factors to the cognitive objectification of sexualized women may not only influence visual body processing in both women and men, but also exacerbate BIC and eating disorders symptomatology in women. For instance, social media continuously portray women in sexualized and piecemeal ways (Conley & Ramsey, 2011; Reichert & Carpenter, 2004) and a longer exposure to visual media has been related to a deeper internalization of westernised beauty ideals (e.g., ‘fitspiration’ and ‘thinspiration’). While both women and men are recipients of sociocultural messages regarding body appearance ideals, for example, thinness-ideal for women and muscular-ideal for men within Westernized cultures (Cafri et al., 2005; Thompson, Heinberg, Altabe, & Tantleff-Dunn, 1999), women may be more often targeted and more negatively impacted than men (see for e.g., Bearman, Presnell, Martinez, & Stice, 2006). This, in turn, may impact negatively on the body image of women more than men (e.g., Lanis & Covell, 1995; Milburn, Mather, & Conrad, 2000).

In sum, while previous studies have provided evidence for the specialised processing of others’ faces and bodies, the relative role of the observer’s and model’s gender and their interaction remains ambiguous. Furthermore, no studies so far have identified those socio-cultural factors that may impact on body and face visual processing in women and men. Disentangling the perceptual ability of women and men in discriminating same or opposite sex bodily and facial cues, and associated individual differences in beauty ideals may have potentially relevant implications for the understanding and treatment of body image disturbances in patients with an eating disorder. This is especially important considering that a series of studies report perceptual size distortions to generalise to both self and other bodies (Cornelissen, Bester, Cairns, Tovée, & Cornelissen, 2015, Cornelissen, Gledhill, Cornelissen, & Tovée, 2016; Cazzato, Mele, & Urgesi, 2014a, Cazzato, Mian, Serino, Mele, & Urgesi, 2014b but see Thaler et al., 2018 for the opposite result).

## The current study

The present study aims to shed light on the visual processes associated with the perception of female and male bodies in women and men, by using an inversion paradigm. Given that the ‘inversion effect’ has been taken as an indirect indicator of configural processing in an array of studies adopting several stimuli and tasks, we took advantage of the use of a matching-to-sample task (see for e.g., Urgesi et al., 2012, 2014, but also Groves et al., 2019), in which we asked women and men to discriminate same- or opposite-gender bodies that could be presented either upright or inverted. Furthermore, we extended our investigation to faces and control stimuli (a familiar object, i.e., a Coca Cola bottle), to ascertain whether any gender difference relates to visual strategies in perceiving the body only or if it also extends to non-corporeal objects. Given the importance of the internalization of beauty ideals (i.e., thinness and muscularity ideals) in the development of body image and eating disturbances (see Ata, Schaefer, & Thompson, 2015; Thompson & Stice, 2001 for a review), we also asked whether gender differences in the visual processing of female and male bodies are associated with societal and interpersonal aspects of beauty ideals as well as disordered eating traits. To this aim, two self-report scales were administered, namely ‘The Sociocultural Attitudes Towards Appearance Questionnaire’ (SATAQ-4, Heinberg, Thompson, & Stormer, 1995), as a measure of the assessment of sociocultural risk factors for body dissatisfaction and eating pathology, and the ‘Eating Attitude Test-26′ (EAT-26, Garner, Olmsted, Bohr, & Garfinkel, 1982), as a measure of symptoms and concerns characteristic of eating disorders.

We expected that both women and men would show evidence of configural processing of faces and bodies, but not of objects. However, the relative amount of the inversion effect for body and face stimuli might vary according to either the observer’s or model’s gender. In keeping with a gender difference in configural vs. local processing (Cazzato et al., 2014a, b; Groves et al., 2019), we predicted that women would show a greater reduction of the inversion effect (i.e., deficit in configural processing) than men for both male and female bodies and faces. Conversely, in keeping with the ‘Sexualised-body inversion hypothesis’ (Bernard et al., 2012, 2013, 2015; Gervais et al., 2012), we expected that, irrespective of the observer’s gender, female bodies, but not faces, should show weaker inversion effects. Furthermore, given the greater pressure exerted by western societal norms on women than on men (Jones, 2001) and the greater incidence of body image disturbances in women (Striegel-Moore & Bulik, 2007), we predicted that women who report stronger socio-cultural influence, for e.g., higher internalization of the thinness and muscularity ideals and more disordered eating traits than men, also display weaker inversion effects for female body stimuli. Finally, further supported by previous literature showing that individuals suffering from disorders characterised by high-BIC might not process appearance-related bodily stimuli in the typical configural way, but on the basis of their features (Beilharz et al., 2016; Duncum et al., 2016; Feusner et al., 2010a; Mundy & Sadusky, 2014; Urgesi et al., 2012, 2014), thus backing up the notion of a local bias as a marker for BIC (Beilharz et al., 2016), we predicted BIE to correlate with participants’ Body Mass Index (BMI) and scores obtained at the subscales of the SATAQ-4 and of the EAT-26.

## Materials and methods

### Participants

The sample size was based on a preliminary calculation using the freely available G*Power software (G*Power 3.1.9; Faul, Erdfelder, Lang, & Buchner, 2007), which indicated a minimum sample of 44 participants as adequate for a design with 95% power to detect a moderate effect size of the variables (*f* = 0.25), using a mixed design ANOVA with alpha at 0.05 (two tailed). To cope with potential drop out and outlier case exclusion, a total of 48 Caucasian students (women: *n* = 24; mean age = 25.79 years, SD 6.49 years; mean BMI = 24.79 kg/m^2^, SD 5.01 kg/m^2^ and men: *n* = 24, mean age = 24.04 years; SD 5.94 years; mean BMI = 24.13 kg/m^2^, SD 2.73 kg/m^2^, see Table 1) from Liverpool John Moores University (LJMU) participated in both Experiment 1 and Experiment 2 in return for course credits. All subjects but three women and four men were right-handed as assessed by the Edinburgh Handedness Inventory (Oldfield, 1971). Participants were asked to provide their self-identified gender identity (and not their biological sex), which was assessed through two forced-choice boxes (female, male). This choice was made because inferring gender identity from biological sex has been often criticized by current literature (see Mazzuca, Majid, Lugli, Nicoletti, & Borghi, 2020), given that self-determined gender identity does not always match with the sex assigned at birth. Participants (self)reported normal or corrected to normal vision and they were in good health, were free of psychotropic or vasoactive medication, with no current or history of psychiatric or neurological disease. All participants signed a written informed consent and were debriefed at the end of the experiment. All procedures were approved by the university research ethics committee of LJMU, in agreement with the ethical standards of the 1964 Declaration of Helsinki.

**Table 1:** Mean and standard deviation (S.D., in brackets), Minimum and Maximum of demographic variables and self-report questionnaire scores for the two groups of women and men, respectively

	Women *N* = 24			Men *N* = 24			Women vs. men
	Mean (St. Dev.)	Min	Max	Mean (St. Dev.)	Min	Max	*t* and *p*
Age	25.79 (6.49)	18	43	24.04 (5.94)	18	35	*t*(46) = 0.97; *p* = 0.335
BMI	24.79 (5.01)	17.18	34.67	24.13 (2.73)	19.06	30.37	*t*(46) = 0.56; *p* = 0.576
*EAT-26*							
Dieting	6.17 (6.81)	0	20	4.96 (6.56)	0	19	*t*(46) = 0.63; *p* = 0.534
Bulimia/Food Preoccupation	1.79 (4.15)	0	18	1.96 (4.20)	0	18	*t*(46) = -0.14; *p* = 0.891
Oral control	1.96 (1.92)	0	8	1.38 (1.95)	0	8	*t*(46) = 1.04; *p* = 0.302
Total score	9.92 (11.01)	0	44	8.29 (11.27)	0	44	*t*(46) = 0.51; *p* = 0.616
*SATAQ-4*							
Internalization—Thin/Low Body Fat	2.88 (0.88)	1.20	4	2.73 (0.72)	1.6	4	*t*(46) = 0.68; *p* = 0.499
Internalization—Muscular/Athletic	2.05 (0.87)	1	4.2	2.75 (0.74)	1.6	5	*t*(46) = -3.01; *p* = 0.004

The data of the two groups were compared by means of independent sample t-test (two-tailed)

*BMI* Body Mass Index, *EAT-26* Eating Attitude test-26, *SATAQ-4* Sociocultural attitudes toward appearance questionnaire

### Experimental stimuli and task

Stimuli were colour pictures depicting whole body postures and faces of virtual characters (Experiment 1) and objects (Experiment 2). Each stimulus was presented upright and upside down. For the body stimuli, we chose two female and two male models (Alyson, Sydney, James and Torno) previously selected amongst six virtual adult body stimuli, created by means of Poser Pro 2010 (e-frontier, Santa Cruz, CA) (see Cazzato et al., 2012 for specific details). The models were depicted as standing against a grey background and were wearing identical underwear black clothing. Each model was displayed in four different postures, from a frontal or three-quarter perspective, respectively. Furthermore, the apparent body weight of each model varied according to four levels of roundness, so that to create moderate to extreme levels of round and thin figures (4 levels: extremely-round, moderate-round, moderate-thin and extremely-thin). Thus, in total we had 4 (2 female and 2 male) models rendered in 4 different postures and in 4 different body size figures, for a total of 64 stimuli (32 female and 32 male bodies). We did not remove the models’ face since previous studies have shown absent inversion effects for headless bodies (Minnebusch, Suchan, & Daum, 2009; Yovel et al., 2010), but it was scrambled to rule out the impact of face identity discrimination in performing the body inversion task.

Like the body stimuli, virtual facial stimuli were created by means of Poser Pro 2010. Face stimuli were also standardised by asking an independent sample of participants (*n* = 54, 39 women; Mean age = 30.71 years, SD 9.96 years) to provide judgements of gender, roundness and attractiveness of each face. From a database of six-dimensional adult face stimuli, two female and two male faces (Jessi, Sydney, James and Ryan) composed the final set of stimuli. Similar to the body stimuli, the faces were depicted against a grey background and hair were removed to avoid any confound on perceived attractiveness. Each model face was rendered with two neutral expressions and was viewed from a frontal or three-quarter perspective. Furthermore, the apparent weight of the faces was manipulated so to depict moderate to extreme levels of round and thin faces (4 levels: extremely-round, moderate-round, moderate-thin and extremely-thin). Thus, similarly to the body stimuli, we had 4 (2 female and 2 male) face models rendered in 4 different postures and in 4 different size figures, for a total of 64 stimuli (32 female and 32 male faces).

In line with previous studies that have used a bottle as control object for body processing in healthy individuals (Glauert, Rhodes, Byrne, Fink, & Grammer, 2009; Mohr, Porter, Benton, 2007; Cazzato et al. 2014a, b), for Experiment 2 we used object stimuli that depicted pairs of two different virtual exemplars of a coke bottle (e.g., Coca Cola Normal and Coca Cola Light), from a frontal or side perspective and rendered against a grey background. For each exemplar, the apparent weight was set to two different levels to create two levels of round and thin bottles (2 levels: extremely-round, extremely-thin). Furthermore, to increase stimuli variability, per each of the two coke bottles, exemplars were presented both in their original and flipped versions. Thus, we had two Coke bottle exemplars (Normal and Light), 2 sizes (Round and Slim), 2 view (frontal, lateral) by 2 labels (original and flipped) by 2 orientation (upright and inverted) for a total of 32 coke bottle stimuli.

**Fig. 1:**
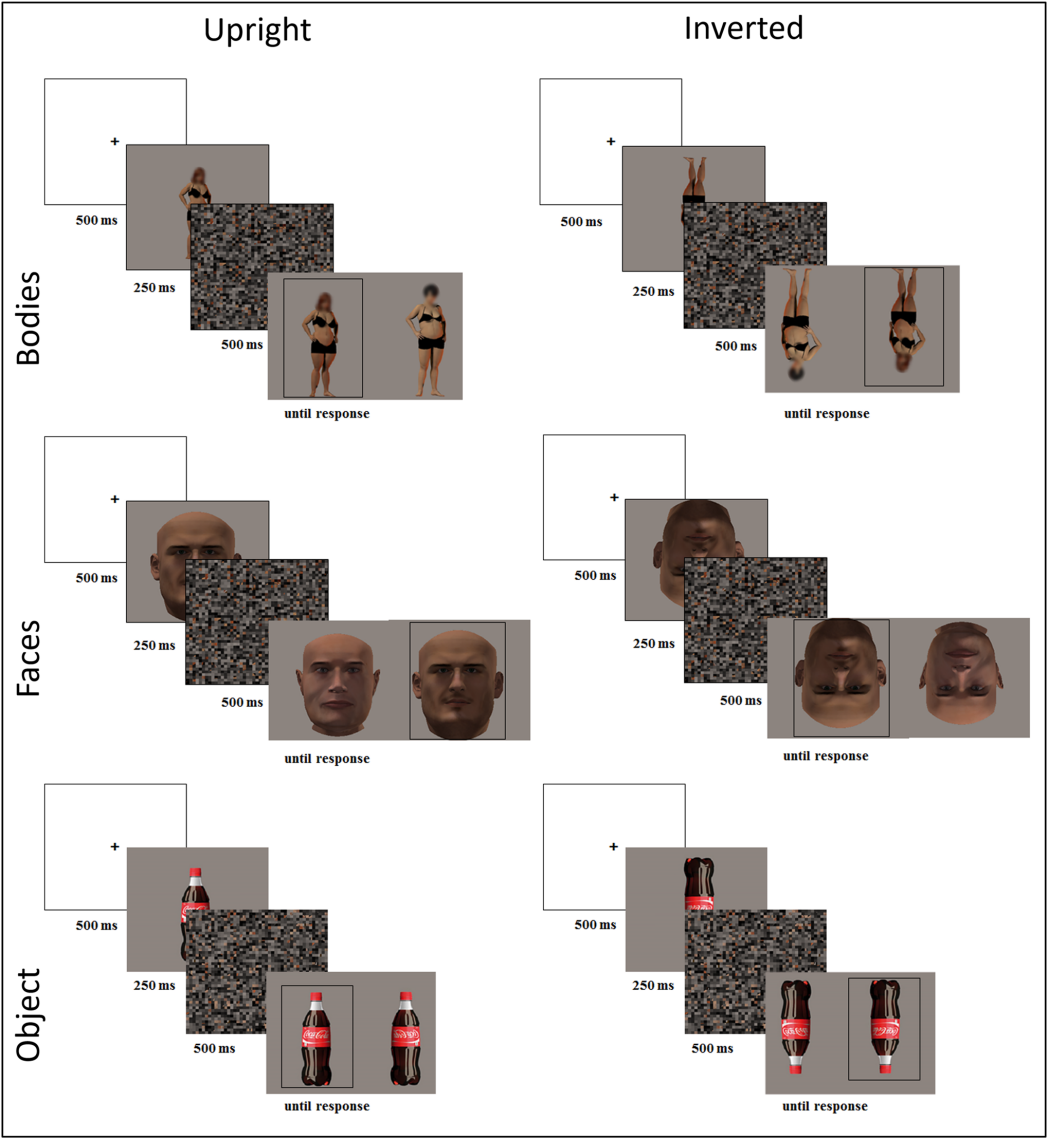
Time course and example of stimuli of the discrimination task trials. One example trial is provided for each experimental condition of the two experiments (Experiment 1: bodies and faces; Experiment 2: objects) and Orientation (upright, inverted). During Experiment 1 both male and female body and face stimuli were presented, respectively

Participants were required to complete a delayed matching-to-sample task adapted from previous studies (Urgesi et al., 2007, 2014) that has shown differences in cortical pathways for visual body processing and the performance of patients with anorexia nervosa and controls in the processing of body, face, and object parts. In this task, participants were asked to choose which of two different probe images was the match for a previously presented sample stimulus. During Experiment 1, in the discrimination of body stimuli, the matching and non-matching stimuli depicted the whole body (with blurred face) of two different models of the same gender and with the very same body posture; in the discrimination of face stimuli, the matching and non-matching stimuli depicted the face of two models of the same gender and with the very same facial expression. During Experiment 2, for the discrimination of object stimuli, the matching and non-matching stimuli depicted two different exemplars of bottles viewed from the same perspective. Thus, for all categories of stimuli, participants were expected to process the morphological cues of the stimuli and to correctly match the correct probe with the sample, since posture, position and view angle were the same in the matching and non-matching stimuli. In each trial, both sample and probe stimuli were presented upright or inverted (see Fig. 1, for an example of body, face and object trials).

### Task procedure

While completing the delayed matching-to-sample task, participants sat approximately 57 cm in front of a 15.6‐inch LCD monitor (resolution, 1024 × 768 pixels; refresh frequency, 60 Hz), where stimuli appeared on a grey background. The experiment was generated via E-Prime software (version 2.0, Psychology Software Tools, Inc., Pittsburgh, PA) running on a PC. In Experiment 1, the body, and face stimuli were presented in two separate 128-trial blocks, with each stimulus presented twice. Like Experiment 1, in Experiment 2, objects were presented in a 128-trial block, with each stimulus presented four times. In each block, 64 upright and 64 inverted trials were randomly presented. The order of the experiments and blocks was counterbalanced between-participants. For each experiment, before the start of each experimental block, participants were introduced to the task and presented with eight practice trials (taken from a different pool of body, face and object stimuli to avoid familiarisation), which were not considered in the main analyses. Participants could rest between blocks for how long they needed, usually no longer than 60 s.

At the beginning of each trial, a 500 ms central fixation point, followed by a sample stimulus presented for 250 ms, was presented at the centre of the monitor. The sample duration was chosen in keeping with previous studies testing body inversion in healthy individuals (Minnebusch et al., 2009; Reed et al., 2006; Yovel et al., 2010), so to ensure the optimal conditions to detect it. A random-dot mask obtained by scrambling the corresponding sample stimulus by means of custom-made image segmentation software (Matlab 9.5, The Mathworks, Inc., Natick, MA, USA) was also presented to limit image persistence (7.6° × 7.6° in size; duration, 500 ms). As soon as the mask disappearance, the two probe stimuli appeared and remained on the screen until a response was given. Stimuli were presented one to the left and one to the right of the screen with 1.5° eccentricity. The presentation of the matching stimulus, to the left or to the right position of the screen was randomized. By using their index finger to press the left or the right key, respectively, participants were instructed to use the mouse and to respond as quickly and accurately whether the matching probe stimulus was the one to the left or to the right of the screen. Participants responded using their dominant hand.

### Self-report questionnaires

After completion of the experimental tasks, participants filled the EAT-26 (Garner et al., 1982), and the SATAQ-4 (Schaefer et al., 2015) questionnaires. Furthermore, participants’ Body Mass Index (BMI; kg/m^2^) was obtained from measuring weight (kg) and height (cm), by means of a digital scale (OMRON BF511) and a stadiometer.

The EAT-26 (Garner et al., 1982) is a 26-item measure of behaviours and cognitions associated with anorexia, bulimia, and binge eating disorder. Items are scored on a 0–3 scale and summed for the global symptom score, with a score of 20 or more indicating a probable eating disorder (King, 1991). The scale comprises three subscales: Dieting, Bulimia and Food Occupation, and Oral Control. Scores for each subscale are calculated by summing the scores across items on that subscale. The EAT-26 has demonstrated good reliability and validity in both women and men (Gleaves, Pearson, Ambwani, & Morey, 2014).

The SATAQ-4 (Schaefer et al., 2015) requires participants to rate their agreement with 22 statements using a 5-point Likert-type scale from 1 (“definitely disagree”) to 5 (“definitely agree”), which are averaged to derive mean scores. It comprises five subscales: thin-ideal internalization, muscularity-ideal internalization, and three pressure subscales (Pressure from media, Pressure from family, Pressure from peers; note that for the purpose of this investigation only the two internalisation subscales were considered). The five items of the thin-ideal internalization subscale assess the endorsement and acceptance of a thin-ideal. The five-items for the muscularity-ideal internalization subscale assess the endorsement and acceptance of a muscular/athletic ideal. Psychometric evaluations of the SATAQ-4 reported evidence of convergent validity with various eating disorders and body dissatisfaction measures, as well as high internal consistency scores in samples of English speaker (Schaefer et al., 2015; Yamamiya et al., 2016).

### Data handling

All statistical analyses were performed using STATISTICA 8.0 (StatSoftInc, Tulsa, Oklahoma). To minimise the impact of speed/accuracy trade-offs, performance was calculated using inverse efficiency (IEs) scores (see Jacques and Rossion, 2007). Expressed in ms, IEs are defined as mean response times (correct responses only) divided by the proportion of correct responses, thus allowing for a combined measure of both accuracy and speed. Higher scores indicate poorer performance in visual discrimination of bodies, faces or objects. IEs were calculated for each condition and for each participant. Reaction Times (ms) and Accuracy (%) are also reported in Table 2 (Experiment 1) and in Table 4 (Experiment 2) for sake of completeness.

**Table 2:** Mean (and s.e.m. in brackets) Reaction times (in ms) and Accuracy (%) of participants as a function of stimuli’ category (bodies, faces), orientation (upright, inverted) and stimuli’ gender (Female, Male)

	Bodies		Faces	
	Female	Male	Female	Male
*Reaction times (ms)*				
Upright	743.66 (27.64)	714.64 (28.98)	656.84 (23.71)	774.07 (26.37)
Inverted	842.27 (34.87)	791.04 (37.80)	709.84 (31.58)	866.40 (33.90)
*Accuracy (%)*				
Upright	92.63 (1.45)	94.85 (1.16)	95.98 (0.73)	93.19 (0.95)
Inverted	86.72 (1.63)	91.53 (1.61)	93.24 (0.85)	85.00 (1.15)

The data from Experiment 1 were analysed by means of a 4-way 2 × 2 × 2 × 2 mixed-model ANOVA with Stimulus Category (Bodies, Faces), Orientation (Upright, Inverted) and Models’ gender (Female, Male) as within-subject factors and Participant’s gender (Women, Men) as a between-subject factor. Furthermore, for the control Experiment 2, a 2-way 2 × 2 mixed-model ANOVA with Orientation (Upright, Inverted) as within-subject factor and Participant’s Gender (Women, Men) as a between-subject factor was performed on IEs obtained from the object inversion task. All data are reported as Mean (M) and Standard Error of the Mean (S.E.M.). A significance threshold of *p* < 0.05 was set for all effects and effect sizes were estimated using the partial eta square measure (*ηp*^2^). Tukey post-hoc tests were performed to follow-up significant interactions. Furthermore, indexes of body (BIE), face (FIE) and object (coke bottle) inversion effect (OIE) were calculated by subtracting, for each category, the IEs in discriminating inverted stimuli from that in discriminating upright stimuli. Overall, the three indexes provide information of the extent to which participants were better at discriminating upright vs. inverted stimuli for bodies, faces and objects, respectively. Finally, to investigate the unique associations between the amount of BIE and FIE for female and male stimuli and participant's BMI, EAT-26 (tot-score) and SATAQ-4 thin- and muscularity-internalization beauty ideals, a series of multiple linear regressions were fit to predict all measures of inversion effect.

## Results

### Demographics and self-report scales’ differences between men and women

The demographics and self-report questionnaire scores of the two groups of women and men are reported in Table 1. An independent sample *t* test indicated that women and men were matched for age, BMI and self-report disordered eating scores as measured by the EAT-26. In line with current literature, men displayed higher scores for the internalization-athlete SATAQ-4 subscale compared to women, indicating significantly stronger internalization of a muscular physique. Finally, although women tended to display higher scores than men at the thin-internalization subscale of the SATAQ-4, no significant difference was found.

### Experiment 1

**Fig. 2:**
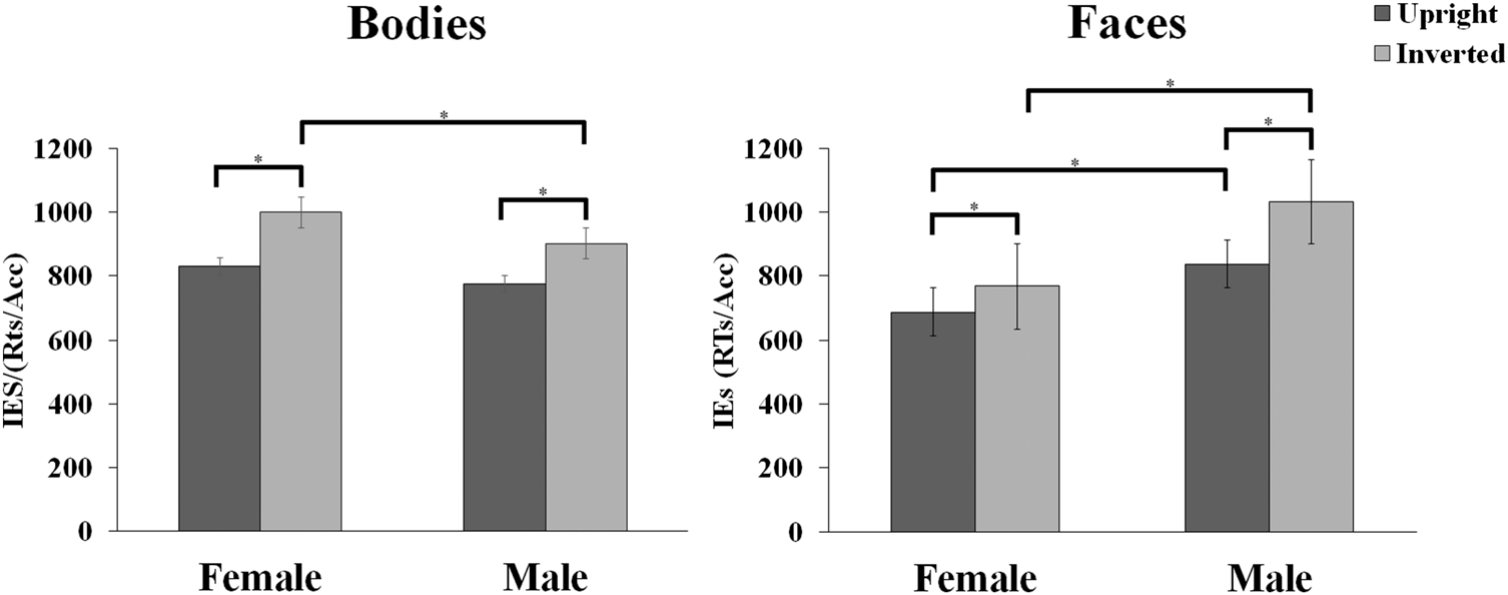
Participants’ performance during Experiment 1. Mean Inverse Efficiency scores (IEs) of participants as a function of stimuli’ category (bodies, faces), orientation (upright, inverted) and stimuli’ gender (Female, Male). Error bars indicate standard errors of the means. Asterisks indicate significant pair-wise comparisons

The analysis of the IEs in the discrimination of upright and inverted body and face stimuli revealed non-significant main effects of Participant’s Gender [*F*_(1,46)_ = 0.186, *p* = 0.668, *ηp*^2^ = 0.004] nor of Stimulus Category [*F*_(1,46)_ = 1.049, *p* = 0.311, *ηp*^2^ = 0.022], but a significant main effect of Stimulus Orientation [*F*_(1,46)_ = 51.884, *p* < 0.001, *ηp*^2^ = 0.530] and of Model’s Gender [*F*_(1,46)_ = 15.518, *p* < 0.001, *ηp*^2^ = 0.252]. These main effects were further qualified by a significant 2-way interaction between Stimulus Category and Model’s Gender [*F*_(1,46)_ = 73.987, *p* < 0.001, *ηp*^2^ = 0.617], and by the significant 3-way interaction of Stimulus Category, Model’s Gender and Stimulus Orientation [*F*_(1,46)_ = 37.68, *p* < 0.001, *ηp*^2^ = 0.45, see Fig. 2).

Tukey post-hoc analyses of this 3-way interaction showed that participants presented a significant BIE (i.e., IEs inverted > IEs upright) for both female (999.98 ms ± 55.56 ms vs. 829.60 ms ± 48.73 ms, *p* < 0.001) and male body stimuli (902.06 ms ± 61.71 ms vs. 775.11 ms ± 48.93 ms, *p* < 0.001). Such inversion effects were more prominent for female bodies (mean difference = 170.38 ms) than for male bodies (mean difference = 126.96 ms). For upright conditions, efficiency in discriminating female bodies was comparable to that of male bodies (*p* = 0.165). On the opposite, for inverted orientation conditions, the discrimination efficiency for female bodies was significantly lower than that for male bodies (*p* = 0.001).

Regarding the face inversion effect, participants presented a significant FIE (i.e., IEs inverted > IEs upright) for both female (767.77 ms ± 37.34 ms vs. 688.04 ms ± 27.02 ms, *p* = 0.008) and male face stimuli (1032.13 ms ± 46.97 ms vs. 837.29 ms ± 32.96 ms, *p* < 0.001). Such inversion effects were more prominent for male (mean difference = 194.84 ms) than for female faces (mean difference = 79.73 ms). For upright conditions, efficiency in discriminating male faces was lower compared to that in discriminating female faces (*p* < 0.001). Similarly, for inverted orientation conditions, efficiency in discriminating male faces was lower compared to that for female faces (*p* < 0.001).

Finally, whilst efficiency in discriminating inverted female stimuli was lower for the body than for the face (999.98 ms ± 55.56 ms vs. 767.77 ms ± 37.34 ms, *p* < 0.001), the efficiency in discriminating inverted male stimuli was lower for the face than for the body (1032.13 ms ± 46.97 ms vs. 902.06 ms ± 61.71 ms, *p* < 0.001). Analogously, discriminating upright female stimuli was significantly less efficient for bodies than faces (829.60 ms ± 48.73 ms vs. 688.04 ms ± 27.02, *p* < 0.001). On the contrary, no difference in performance was found for male body and face stimuli when presented upright (775.11 ms ± 48.93 ms vs. 837.29 ms ± 32.96 ms, *p* = 0.072).

Only the linear multiple regression analysis calculated to predict the BIE for male stimuli from BMI, eating concerns and beauty ideals scores was significant [*F*_(4,43)_ = 2.633, *p* = 0.047, *R*^2^ = 0.44], with BMI and internalisation of muscularity (SATAQ-4) emerging as significant predictors (see Table 3).

**Table 3:** Standardized coefficients from the linear model multiple regression of BMI, eating concerns and beauty ideals predicting the amount of body inversion effect (BIE) and face inversion effect (FIE) for male and female stimuli, respectively

	*B*	SE	*β*	*t* value	*p* level
BIE					
Male					
BMI	0.30	0.14	18.84	2.20	0.033
EAT-26 Total score	0.06	0.15	1.29	0.38	0.704
SATAQ internalization-thin/low body fat	− 0.09	0.17	− 27.41	− 0.52	0.607
SATAQ internalization-muscular/athletic	0.37	0.16	104.73	2.32	0.025
Female					
BMI	0.07	0.15	2.24	0.46	0.65
EAT-26 Total Score	− 0.06	0.16	− 0.66	− 0.34	0.73
SATAQ internalization-thin/low body fat	− 0.10	0.19	− 16.47	− 0.54	0.59
SATAQ internalization-muscular/athletic	− 0.06	0.17	− 8.44	− 0.33	0.75
FIE					
Male					
BMI	0.14	0.15	6.89	0.95	0.35
EAT-26 Total score	− 0.03	0.16	− 0.50	− 0.17	0.86
SATAQ Internalization—thin/low body fat	0.25	0.18	63.45	1.41	0.17
SATAQ Internalization-muscular/athletic	0.01	0.17	1.71	0.04	0.96
Female					
BMI	0.16	0.15	5.19	1.09	0.28
EAT-26 Total score	0.04	0.16	0.51	0.27	0.79
SATAQ Internalization-thin/low body fat	0.07	0.18	11.61	0.39	0.70
SATAQ Internalization-muscular/athletic	0.08	0.17	11.00	0.44	0.66

*BMI* Body Mass Index, *EAT-26* Eating Attitude test-26, *SATAQ-4* Sociocultural attitudes toward appearance questionnaire

### Experiment 2

The 2-way ANOVA on IEs for the object stimuli revealed only a significant main effect of Orientation [*F*_(1,46)_ = 12.223, *p* = 0.001, *ηp*^2^ = 0.210], which was due to participants being less efficient in discriminating objects when presented inverted as compared to upright (1348.66 ms ± 53.31 ms vs. 1203.65 ms ± 60.46 ms, *p* < 0.001). No other effects were significant [All *Fs* < 2.291, p > 0.137, *ηp*^2^ < 0.047]. Mean RTs, Accuracy and IEs are reported in Table 4. Importantly, no significant associations between the IEs of the participants’ OIE and their BMI, and individual scores at the EAT-26 and SATAQ-4 subscales were found (see Table 5).

**Table 4:** Participants’ performance during Experiment 2 (object)

	Upright	Inverted
Reaction times (in ms)	1026.67 (38.65)	1119.90 (41.63)
Accuracy (%)	87.47 (1.66)	84.27 (1.66)
IEs	1203.65 (60.46)	1348.66 (53.31)

Mean (and s.e.m. in brackets) Reaction times (in ms), Accuracy (%) and Inverse Efficiency scores (IEs) as a function of the object’s Orientation (upright, inverted)

**Table 5:** Standardized coefficients from the linear model multiple regression of BMI, eating concerns and beauty ideals predicting the amount of object inversion effect (OIE)

Objects	*B*	SE	*β*	*t* value	*p* level
BMI	0.00	0.15	0.26	0.02	0.98
EAT-26 Total Score	− 0.07	0.17	− 1.77	− 0.41	0.69
SATAQ Internalization-thin/low body fat	0.04	0.19	13.66	0.20	0.84
SATAQ Internalization-muscular/athletic	0.11	0.17	35.87	0.62	0.54

*BMI* Body Mass Index, *EAT-26* Eating Attitude test-26, *SATAQ-4* Sociocultural attitudes toward appearance questionnaire

## Discussion

In the current study, we sought to investigate the perceptual ability of women and men in the configural and feature-based processing of female and male bodies and faces. Furthermore, we asked whether gender effects in configural and local processing of body and face stimuli were associated to BMI, societal beauty ideals and traits of disordered eating. We found that regardless of participants’ genders, female and male bodies and faces yielded typical inversion effects. Thus, discriminations of bodies and faces were significantly better for upright than for inverted bodies and faces. Contrary to our hypothesis, women's processing strategies for female bodies did not vary with their degree of self-reported thinness ideal internalization and disordered eating traits. Furthermore, we did found evidence of object inversion effect, again regardless of the observer’s gender or individual traits, which likely reflects the high familiarity of the object. Indeed, the use of configural processing is greater for more familiar stimuli (e.g., Campbell & Tanaka, 2018), such that domain-specific expertise with a class of objects, for example words, dogs, birds or even artificial objects may lead to face-like perceptual processing and reliable inversion effects (Wong, Palmeri, & Gauthier, 2009; Wong, Wong, Lui, Ng, & Ngan, 2019; Diamond & Carey, 1986; Campbell & Tanaka, 2018). Importantly, this object inversion effect was not modulated by the observer’s gender and was not associated to any of the self-report scales measuring BMI, eating disorders symptomatology or internalization of beauty ideals.

Regarding the effects of gender, however, we found that the between-subjects effect of participants’ gender was not significant nor did it interact with any of the within-subjects factors also for the discrimination of bodies and faces. Thus, contrary to our expectations, the use of configural processing was not modulated by the observer’s gender. On the other hand, what seemed to play a major role in affecting participants’ performance was the gender of the stimuli. With this regard, participants showed an overall greater disadvantage after inversion of female compared to male bodies. In fact, for the upright condition, efficiency in discriminating female bodies was comparable to that of male bodies; in contrast, when the stimuli were presented inverted, discrimination performance was better for male than female bodies, thus pointing to a preference in local processing of male bodies. The opposite was observed for the discrimination of faces, with a greater disadvantage after the inversion of male compared to female faces. This effect supports the notion that that female and male bodies and faces are processed in distinct ways, with greater bias towards configural processing of female than male bodies and of male than female faces. Thus, whilst women and men adopt very similar visual strategies when analysing the body of conspecifics, they do seem to rely to a different extent on configural vs. local processing depending on the gender of the bodies.

The finding of reliable inversion effects independently from the observer’s gender is in keeping with the findings Groves et al. (2019), who found typical configural face and body processing in both gender groups, even though, differently from our study, the BIE was partially reduced in young female participants at risk of developing an eating disorder. In a similar vein, a study from Urgesi et al. (2014) found a deficit in configural body processing in (female only) patients with AN compared to controls. In contrast with these findings, our regression analysis did not reveal any relationship between eating disorder traits and use of body or face configural processing. It is worth noting, however, that in our study, there was essentially no evidence for presence or high risk for body image disturbances and eating disorder traits, as measured by the global score of the EAT-26. Specifically, participants scored below the average of 9.92 (clinical cut-off = 20), which do not ‘categorise’ our sample as particularly at risk for developing eating disorders (and participants with previous or current history of any psychiatric disorders were also not eligible for the study). Therefore, it is possible that the lack of a bias towards local processing for body stimuli as observed in anorectic patients in previous study did not occur in our sample because overall levels of eating disorders symptomatology were within the normal range.

Importantly, while the observer’s gender did not matter, the gender of the observed body played a major role in influencing to what extent both female and male observers used configural processing of female or male bodies and faces. While the effect of the model’s gender is in keeping with the ‘Sexualised-body inversion hypothesis’ (Bernard et al., 2012, 2013, 2015), its direction is opposite. Indeed, a reduced use of configural processing of female than male bodies was expected by this view, while we obtained exactly the opposite results, with greater inversion effects for female than male bodies. In other words, whilst our participants valued more the appearance of single body parts of male models, thus relying more on local than configural processing, the opposite might hold true for discrimination of female bodies, as body processing in this case relied more on the whole-body shape than on feature-based processing. One likely reason for this discrepancy is the type of experimental stimuli used. Indeed, previous studies have shown that the reduced use of configural processing of a female body occurs only for sexualized representations of it (see Bernard et al., 2012; Bernard, Content, Deltenre, & Colin, 2018), scarcely dressed and in sexualized poses. Although also our bodies were wearing only underwear, they were (computer-generated) female and male full bodies in neutral postures, in keeping with the type of non-sexualised stimuli used in studies that have not reported evidence of local processing bias for female bodies (Groves et al., 2019).

However, to the best of our knowledge, we provide first evidence to suggest that both women and men display a preference towards local processing when discriminating male bodies. Indeed, we found that inverting male body stimuli impaired discrimination performance to a less extent compared to inverting female bodies. This might suggest that, at least in our stimulus set, male body stimuli contained clear diagnostic (sexual) information for body attractiveness, which are likely to receive detailed visual inspection in body processing, thus explaining why we observed a reduction of BIE for the male bodies. Indeed, to cover primary sex-typing features, female bodies were covered in both the upper and lower body half, while men were covered only in the lower body half, leaving the upper torso undressed. Thus, our stimuli may have triggered a greater objectified processing of male than female bodies. Crucially, lower and upper body half seems to play a different role for the judgements of the attractiveness of female and male bodies, respectively. Indeed, women tend to prefer men whose torso have the shape of an inverted triangle, that is a narrow waist and a broad chest and shoulders, in keeping with physical strength and muscle development in the upper body and with the societal internalization of muscularity ideals. Accordingly, a study investigating characteristics of male attractiveness for women showed that the waist-chest ratio (WCR, a measure of upper body shape) but not the waist-hip ratio (WHR, a measure of lower body shape) is the most important predictor for woman’s ratings of male attractiveness (Tovée, Maisey, Emery, & Cornelissen, 1999a; Tovée, Maisey, Vale, & Cornelissen, 1999b; Swami et al., 2007).

Interestingly, the bias toward a local processing of male bodies was attenuated for those observers that had greater BMI and greater internalization of muscularity as a body ideal. Previous research has indicated that the BMI of an individual is an important factor in how both men and women perceive physical attractiveness (see Singh 1993; Henss, 1995; Furnham, Tan, & McManus, 1997; Tovée, Reinhardt, Emery, & Cornelissen, 1998, Tovée et al., 1999a, b, Tovée, Tasker, & Benson, 2000; Thaler et al., 2018; Tovee & Cornelissen 1999, 2001). Hence, in keeping with these studies, we might expect an impact of BMI on women’s and men’s familiarity with male bodies and, ultimately, with their ability to inspect male bodily features and body morphology useful for sexual selection and mating success.

Another result worthy of attention is the reduction of FIE for female faces as compared to male faces, with both upright and inverted female faces being discriminated better than both upright and inverted male faces. Previous research has well documented the importance of female facial beauty such as facial shape and form, but also facial skin colour distribution (see Fink, Grammer, & Matts, 2006), since these are linked to women’ age and reproductive condition. In agreement with these results, we argue that the skilled local processing of female faces might be linked to the ancestral ability of searching for those facial cues that signal genetic fertility quality in women. This occurs not only for men judging the attractiveness of a woman, but also for other female observers judging the attractiveness of potential rivals. Indeed, from an evolutionary perspective, women and men engage in sexual competition to attract or to retain a mate. When they compare themselves to potential rivals, they attend to the traits that contribute to a woman and man’ mate value (Buss et al., 2000). In relation to this, an interesting study of Fink, Klappauf, Brewer and Shackelford (2014) in female intra-sexual competition reported that the most feminine faces were perceived as threat, and received higher attractiveness ratings than the other (less feminine) faces, a pattern which was not found with other bodily cues such as breast size and body shape (i.e., WHR). Thus, the local processing bias of female faces displayed by both female and male observers may reflect the evolutionary function of discriminating between potential mates or rivals.

Interestingly, a bias towards a more detail-based type of processing of human faces has been described in previous investigations that focussed on patients with body dysmorphic disorder (Feusner, Townsend, Bystritsky, & Bookheimer, 2007, Feusner, Hembacher, Moller, & Moody, 2011; Feusner et al., 2010a, b), and amongst students with body dysmorphic concerns (Mundy & Sadusky, 2014). Although, in the current study we did not include a measure of body dysmorphic concerns such as the Dysmorphic Concern Questionnaire (DCQ, designed as a screening tool for body dysmorphic disorder, Oosthuizen, Lambert, & Castle 1998) as used in previous studies (Mundy & Sadusky, 2014), although speculative, one possibility to explain our results could be ascribed to a continuous scrutinization of others’ face appearance in a featural, piecemeal way which is linked to high body dysmorphic concerns.

Our findings should be viewed considering their limitations. First, because we did not collect information of participants’ sexual orientation, we cannot rule out that other variables than the (binary) gender categorization, such as observers’ sexual preference, may have affected participants’ responses. With this regard, an interesting study from Jiang, Costello, Fang, Huang, and He (2006) used an interocularly suppression paradigm to present nude images while leaving the observer unaware of the presented stimulus. They showed that female nudes captured attention in heterosexual men and, partly, in homosexual women; conversely, male nudes captured attention in heterosexual women, in homosexual men and, partly, in homosexual women. These results suggest that both the gender and the sexual orientation of the observers modulate their attention to body stimuli. In a similar vein, also a mating strategy to prefer short- or long-term relationships may influence the attraction to more feminine or masculine bodies (Bailey, Gaulin, Agyei, & Gladue, 1994). For example, among heterosexual women, those who are more open to short-term relationships tend to prefer masculine male bodies (Provost, Kormos, Kosakoski, & Quinsey, 2006) and faces (Waynforth, Delwadia, & Camm, 2005) to a greater extent than more restricted individuals. Taken together, the results of these studies lend support to the idea that an individual’s gender and sexual orientation are multi-layered conceptual representations lying on a continuum, which cannot be captured by the simplistic dichotomies between biological qualities of the human body and cultural or social aspects of sex expressions (see Mazzuca et al., 2020; Fausto-Sterling, 1993, 2019). Accordingly, although we did not find effects of a dichotomous self-evaluation of gender identity in our participants, this does not rule out that their level of gender identification, sexual orientation and mating strategy may have affected the processing of female and male bodies. Future studies in larger samples are required to acknowledge the full spectrum of human body configurations and clarify their influence on body perception and appreciation.

Furthermore, although the body inversion paradigm examines whether bodies are processed either configurally (i.e., better recognition of upright vs. inverted bodies) or analytically (i.e., upright and inverted bodies are equally well recognized), it does not enable to directly examine analytic processing with respect to body parts recognition specifically. Future studies need to provide converging evidence using other paradigms, such as the part/whole paradigm to further investigate the role of the observer’s and model’s gender in the configural vs. local processing of bodies and faces parts. Furthermore, though only (self-reported) healthy participants were included, we did not carry out a structured assessment of any prior or current eating disorders (or other type of psychiatric disorders such as body dysmorphic disorder). Although this possibility is unlikely due to the low scores obtained at the EAT-26 by both gender groups, it may still be plausible that undiagnosed (clinical) populations might have contributed to this particular pattern of results. In a similar vein, in the current study we did not record information of follicular and luteal phases of women’ menstrual cycles that could have affected the attentional focus towards masculinity characteristics in male bodies and faces (Frost, 1994; Johnston, Hagel, Franklin, Fink, & Grammer, 2001; Penton-Voak & Perrett, 2000; Penton-Voak et al., 1999, but see Jünger, Kordsmeyer, Gerlach, & Penke, 2018). Thus, further studies are needed to disentangle the relationship between local biases in discriminating male bodies and change across the menstrual cycle in women.

Finally, although several studies have employed a similar familiar, non-corporeal object (Coke bottle) to account for the possibility that visual strategies are generalisable to objects (non-biological) other than the body/face (biological stimuli, see Mohr et al., 2007; Glauert, et al., 2009), yet it might be possible that this type of stimulus may be a trigger of eating concerns in people with BIC and/or eating disorders. Most importantly, a different type of object stimuli other than the coke bottle, such as ‘feminine vs. masculine shoes’ could be used in future investigations so to better address the question regarding specificity for biological vs non-biological stimuli and relative gender differences in visual strategies, as reported in the current investigation.

In conclusion, this was the first study to show that the gender of stimuli may influence configural body processing. Also, this study has enhanced the current research literature by showing that selective reduction of configural body and face processing may occur in women and men possibly because of a general attention to details needed in elaborating visual information important for discriminating salient bodily (perhaps sexual) cues for mate selection and successful reproduction. Furthermore, preferences for features-based elaboration of body and face seem to be influenced by body cues important for physical attractiveness, such as BMI, and are linked to sociocultural influences for the internalization of beauty ideals. In future, behavioural and neuroimaging investigations should be conducted not only to replicate our findings in a larger and clinical sample of individuals suffering from eating disorders or body dysmorphic disorder, but also to deepen our understanding of the different neurocognitive processing of female and male bodies and faces.

## Supplementary Information

Below is the link to the electronic supplementary material.

Supplementary file1 (XLSX 32 KB)

## Data Availability

The datasets generated during and/or analysed during the current study are available in the form of supplemental materials.
